# Shape-induced superstructure formation in concentrated ferrofluids under applied magnetic fields^1^

**DOI:** 10.1107/S1600576722010093

**Published:** 2022-12-01

**Authors:** Philipp Bender, Erik Wetterskog, German Salazar-Alvarez, Lennart Bergström, Raphael P. Hermann, Thomas Brückel, Albrecht Wiedenmann, Sabrina Disch

**Affiliations:** a Heinz Maier-Leibnitz Zentrum (MLZ), Technische Universität München, Germany; b Department of Materials and Environmental Chemistry, Stockholm University, 106 91 Stockholm, Sweden; c Ångström Laboratory, Department of Materials Science and Engineering, Uppsala University, 751 03 Uppsala, Sweden; d Center for Neutron Scattering, Uppsala University, 751 20 Uppsala, Sweden; e JCNS-2, PGI-4, Forschungszentrum Jülich, Germany; f Materials Science and Technology Division, Oak Ridge National Laboratory, Tennessee, USA; g Institut Laue–Langevin, Grenoble, France; h Department of Chemistry, Universität zu Köln, 50935 Köln, Germany; University of Luxembourg

**Keywords:** ferrofluids, nanocubes, dipolar interactions, small-angle neutron scattering, magnetic SANS

## Abstract

The field-induced ordering of concentrated ferrofluids based on spherical and cuboidal maghemite nanoparticles is studied using small-angle neutron scattering, revealing a qualitative effect of the faceted shape on the interparticle interactions as shown in the structure factor and correlation lengths. Whereas a spatially disordered hard-sphere interaction potential with a short correlation length is found for ∼9 nm spherical nanoparticles, nanocubes of a comparable particle size exhibit a more pronounced interparticle interaction and the formation of linear arrangements. Analysis of the anisotropic two-dimensional pair distance correlation function gives insight into the real-space arrangement of the nanoparticles. On the basis of the short interparticle distances found here, oriented attachment, *i.e.* a face-to-face arrangement of the nanocubes, is likely. The unusual field dependence of the interparticle correlations suggests a field-induced structural rearrangement.

## Abstract 2

In-field small-angle neutron scattering reveals superstructure formation in a concentrated dispersion of spherical and cuboidal iron oxide nanoparticles.

## 1. Introduction

The response of magnetic nanoparticles to applied static and dynamic magnetic fields is the subject of intense research in view of its fundamental technological importance, *e.g.* for medical applications such as imaging and magnetic hyperthermia (Pankhurst *et al.*, 2003 ▸; Gavilán *et al.*, 2021 ▸) or sensor applications (Chen *et al.*, 2010 ▸; Gloag *et al.*, 2019 ▸). The heating behaviour of magnetic nanoparticles in an AC magnetic field is largely influenced by interactions arising from nanoparticle aggregation (Andreu *et al.*, 2020 ▸). Field-assisted self-organization of shape-anisotropic nanoparticles in dispersions is further desired for liquid crystalline or optically anisotropic materials (Whitesides & Grzybowski, 2002 ▸) and is a pre­requisite for self-organization into long-range-ordered arrangements (Ahniyaz *et al.*, 2007 ▸). A detailed understanding of interparticle interactions in colloidal dispersion is therefore crucial for the advancement of applications in magnetic hyperthermia and beyond.

A variety of nanoparticle arrangements have been observed for colloidal dispersions of spherical magnetic nanoparticles with different particle sizes, ranging from short chain segments (Barrett *et al.*, 2011 ▸) to reversible chain formation (Toulemon *et al.*, 2016 ▸; Nandakumaran *et al.*, 2021 ▸), extended columns (Klokkenburg *et al.*, 2007 ▸; Tan *et al.*, 2018 ▸) including needle-like arrangements of nanoparticles (Mamiya *et al.*, 2021 ▸; Wetterskog *et al.*, 2018 ▸) and even pseudo-crystalline organization of nanoparticles (Wiedenmann *et al.*, 2003 ▸; Fu *et al.*, 2016 ▸). Small-angle scattering techniques are powerful methods to study magnetic nanoparticles and reveal *in situ* the structure formation and orientation of the arrangements formed in applied magnetic fields (Mühlbauer *et al.*, 2019 ▸; Honecker *et al.*, 2022 ▸).

The organized arrangement of magnetic nanoparticles depends on the subtle balance between attractive and repulsive interparticle interactions, including hard core repulsion, van der Waals attraction and magnetic dipole interaction (Bishop *et al.*, 2009 ▸). Therefore, flat nanoparticle surfaces and their enhanced van der Waals interactions play a pivotal role in the self-organization of faceted nanoparticles.

The strong structure-directing influence of particle shape on the symmetry of mesocrystalline arrangements has been studied in detail for nanocubes with varying degrees of corner truncation (Disch *et al.*, 2011 ▸, 2013 ▸; Wetterskog *et al.*, 2016 ▸). The self-organization process of magnetic iron oxide nanocubes in a levitating experiment has been reported to consist of two stages, namely the disordered agglomeration of nanoparticles and their transformation into ordered mesocrystals (Agthe *et al.*, 2016 ▸) where the added hierarchical complexity of oriented alignment of the mesocrystals into microscale mesocrystal fibres is observed under an external field (Kapuscinski *et al.*, 2020 ▸). Complex arrangements such as helical chains and filaments of nanocubes have been obtained by self-organization confined to a liquid interface in moderate magnetic fields up to 200 mT (Singh *et al.*, 2014 ▸, 2015 ▸; Liao *et al.*, 2022 ▸).

Despite all these efforts, the behaviour of ferrofluids based on faceted nanoparticles in strong magnetic fields is far from understood. Here, we give a detailed account of the impact of nanoparticle shape on the interparticle correlations in concentrated colloidal dispersions (>5 vol.%) of ∼9 nm maghemite nanoparticles. Particular emphasis is placed on the agglomeration behaviour of cuboidal nanoparticles in highly concentrated dispersions. Using field-dependent small-angle neutron scattering experiments, in combination with analysis of the two-dimensional pair correlation function, we extract the field-induced agglomeration of the particles and the orientation of the arrangements formed. A peculiar field dependence of the structure function is found, which we interpret as a field-induced structural reorganization.

## 2. Experimental

### 2.1. Synthesis and nanoparticle characteristics

Maghemite (γ-Fe_2_O_3_) nanoparticles with spherical and cuboidal shapes were synthesized by thermal decomposition of iron oleate. Both samples have already been part of earlier studies including magnetization characterization (Disch *et al.*, 2012 ▸), and detailed information on their synthesis, including electron microscopy information, is given by Wetterskog *et al.* (2014 ▸).

The small-angle scattering by both samples follows a pure form factor behaviour in dilute dispersion. The nanospheres are characterized by a particle radius of 4.9 (1) nm, with a log-normal size distribution of σ_log_ = 5.5 (1)%, using small-angle X-ray scattering and small-angle neutron scattering (SANS) measurements. The nanocubes’ morphology was previously described using a spherical form factor with a radius of 5.3 (1) nm, which is in agreement with the cuboidal edge length of 8.6 nm as determined using transmission electron microscopy. The size distribution of σ_log_ = 7.2 (2)% is overestimated due to the choice of a spherical form factor. Determination of the superball geometry revealed a significantly larger edge length of 9.6 nm with a superball shape parameter *p* = 1.4, indicating substantial ageing of the nanocubes (Dresen *et al.*, 2021 ▸). The SANS data presented here, however, were collected on the as-prepared nanoparticles.

Magnetization measurements of both samples have been presented by Disch *et al.* (2012 ▸), yielding nanoparticle moments of 13 500 (50) μ_B_ and 16 650 (50) μ_B_ per particle and spontaneous magnetizations of 260 kA m^−1^ and 300 kA m^−1^ for the nanospheres and nanocubes, respectively.

### 2.2. Nanoparticle stabilization

The nanoparticles under study are stabilized by a protective oleic acid ligand layer and dispersed in d_8_-toluene. To stabilize the highly concentrated dispersions against precipitation, a small amount of additional oleic acid was added. The slightly enhanced incoherent scattering background observed using SANS was subtracted before data analysis.

### 2.3. Small-angle neutron scattering

SANS was performed on the D22 instrument at ILL, Grenoble, France. Nanoparticle dispersions with iron oxide concentrations of 261 mg ml^−1^ (5.29 vol.%, spheres) and 271 mg ml^−1^ (5.49 vol.%, cubes) and dilute dispersions with an iron oxide concentration of 7 mg ml^−1^, each in d_8_-toluene, were measured at ambient temperature with a neutron wavelength of 6 Å. Two instrument configurations were used with detector distances of 2 m and 8 m and a collimation distance of 8 m. A magnetic field of up to 1.54 T was generated using an electro­magnet and applied horizontally, perpendicular to the neutron beam.

Data were calibrated using reference measurements of the solvent and the blocked beam and calibrated to absolute scattering intensities using the *GRASP* software (Dewhurst, 2003 ▸). Sectors of 20° opening angle were applied for radial integration parallel and perpendicular to the applied field direction.

The fractal dimension *f* is extracted from the slope of the scattering intensities as a function of the magnitude of the scattering vector **Q** [*Q* = (4π/λ)sin(θ), where 2θ is the scattering angle and λ is the wavelength of the incident radiation] in a *Q* range of 0.008 Å^−1^ < *Q* < 0.02 Å^−1^ following a mass fractal according to *I*(*Q*) ∝ *Q* ^−*f*^.

### 2.4. Data analysis by the singular value decomposition

The 2D scattering data *I*(*Q*, θ) with *i* = *N* data points are directly related to the 2D pair distance distribution function *P*(*r*, ϕ) via 

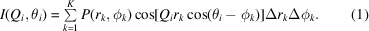

The angle ϕ specifies the orientation of **r** in real space in the *yz* plane. The pair distance distribution function *P*(*r*, ϕ) is directly related to the autocorrelation function γ(*r*, ϕ) via *P*(*r*) = *r*γ(*r*, ϕ) and has characteristic shapes depending on the geometry of the scatterers.

To determine the correlation function with *K* pixels, various approaches exist. In this work we utilize the singular value decomposition (SVD) as described in detail by Bender *et al.* (2019 ▸). Briefly, the matrix **A** with matrix elements *A*_*ik*_ = cos[*q*_*i*_ *r*_*k*_cos(θ_*i*_ − ϕ_*k*_)]Δ*r*_*k*_Δϕ_*k*_ is decomposed according to **A** = **USV** ^T^ into three matrices. The pair distance distribution function can be then computed with *P* = **VS** ^+^ **U** ^T^ **I**, where **S** ^+^ has the reciprocal values 1/*s* of the singular values *s* from the matrix **S** in its diagonal. Very small values of *s* are associated with noise. Thus, to reduce the noise of the solution, small *s* values are systematically eliminated until a reasonable distribution *P* is obtained.

Comparison with the standard indirect Fourier transform shows that the SVD is a fast and robust approach to extract the underlying real-space correlation functions from reciprocal small-angle scattering data (Bender *et al.*, 2022 ▸).

In this work the scattering data had *i* = 8654 points in a *Q* range of 0.008 Å^−1^ < *Q* < 0.2 Å^−1^, we computed pair distance distribution functions with *K* = 17 424 pixels in an *r* range up to *r* = 60 nm, and we used in each case only the ten largest singular values.

## 3. Results and discussion

To reveal the influence of the nanoparticle shape on its agglomeration behaviour in a colloidal dispersion, we investigated concentrated dispersions of maghemite (γ-Fe_2_O_3_) nanospheres and nanocubes with 10 nm diameter and 8.6 nm edge length, respectively, and excellent monodispersity with log-normal size distributions of 5.5 (1)% and 7.2 (2)% FWHM (Disch *et al.*, 2012 ▸). The cuboidal shape of the nanocubes was quantified by a superball shape parameter *p* = 1.4 (1), where *p* = 1 corresponds to a sphere and *p* → ∞ for a perfect cube (Dresen *et al.*, 2021 ▸). The integral nanoparticle moments are very similar for both samples, at 13500 (50) μ_B_ and 16650 (50) μ_B_ per particle, and spontaneous magnetizations of 260 kA m^−1^ and 300 kA m^−1^ for the nanospheres and nanocubes, respectively. As a result of the cuboidal shape, a stronger degree of near-surface spin disorder has been observed for the nanocubes using magnetic SANS, whereas the magnetization in the nanoparticle interior was found to be unaffected by the particle shape (Disch *et al.*, 2012 ▸).

**Figure 1:**
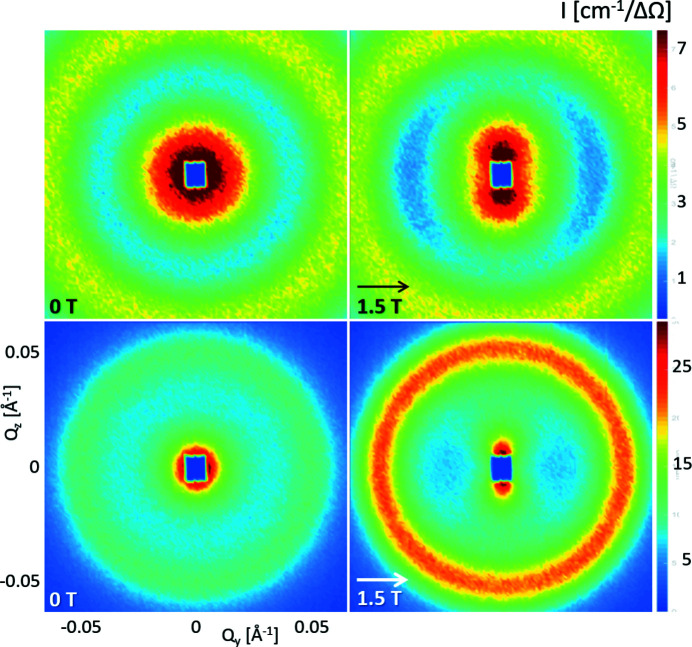
SANS detector images for (top) concentrated nanospheres and (bottom) nanocubes in (left) zero field and (right) a magnetic field of 1.5 T applied in the horizontal direction.

Despite their similar particle size, magnetic moment and volume concentration (>5 vol.%), we observe a significantly distinct agglomeration behaviour of nanosphere and nanocube dispersions using SANS. Both samples reveal a short-range-ordered interaction potential with random orientation towards the applied magnetic field, which is observed as a ring-shaped correlation peak in the 2D SANS detector (Fig. 1 ▸). The interaction potential appears much stronger for the nanocubes, as indicated by the more intense ring and clear field dependence. Additionally, a scattering contribution in the lower *Q* range indicates attractive interparticle interactions that become directionally anisotropic with applied field. In the following, we will analyse both features in the scattering pattern, first in reciprocal space and then using the 2D pair distance distribution function.

### 3.1. Structure factor in concentrated ferrofluids

**Figure 2:**
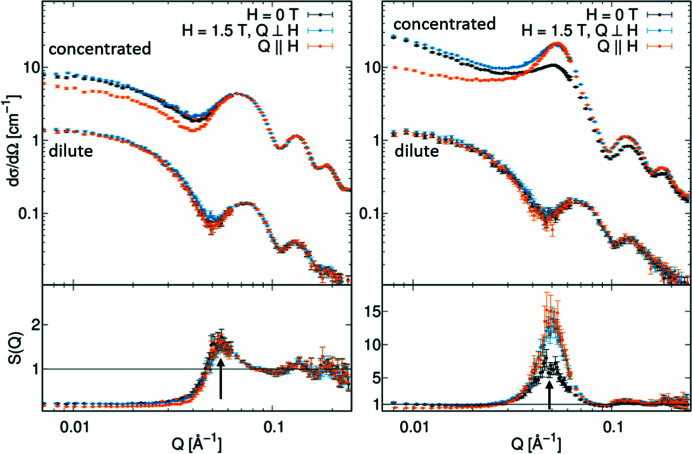
SANS for (left) nanospheres and (right) nanocubes, comparing concentrated (261 and 271 mg ml^−1^, >5 vol.%) and dilute (7 mg ml^−1^, lower scattering cross section) dispersions. Data are shown for the isotropic case (zero field, black) and radially integrated in directions parallel (orange) and perpendicular (blue) to the applied magnetic field of 1.5 T. The bottom panels show the extracted structure factors with the nearest-neighbour correlation peaks (arrows).

In the concentrated dispersions of both iron oxide nanocubes and nanospheres, we observe a clear structure factor indicating significant interparticle interactions, whereas dilute dispersions follow a pure form factor behaviour. The structure factors *S*(*Q*) presented in Fig. 2 ▸ were extracted from the scattering data of the concentrated dispersion [*I*(*Q*)_conc_] and the experimental form factor measured for a dilute dispersion [*I*(*Q*)_dilute_] according to 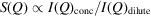 . For both samples, the extracted structure factor exhibits a clear correlation peak corresponding to the mean value of the first nearest-neighbour interaction distance according to *D* = 2π/*Q*.

For the nanospheres, the extracted structure factor is nearly independent of the applied magnetic field, and a nearest-neighbour interaction distance of 11.5 (1) nm is derived from the correlation peak. The nanocube dispersion exhibits a much stronger structure factor with a more intense correlation peak corresponding to an interparticle distance of 12.7 (1) nm. The stronger structure factor observed for the nanocubes is probably induced by the shape anisotropy of the cuboidal particles. Enhanced van der Waals interactions between particle facets commonly induce face-to-face orientation of faceted particles (Disch *et al.*, 2011 ▸) and may therefore, in addition to field-induced dipolar interactions, lead to enhanced interparticle interactions compared with the nanospheres. Relative to the inorganic particle core sizes, the determined interaction distances are surprisingly short, leaving only small separation distances between the iron oxide surfaces of adjacent particles of 2.5 nm for the nanospheres and 4.2 nm for the nanocubes. In particular for the nanospheres, this interparticle distance is small, considering the oleic acid ligand shell stabilizing the nanoparticles. The oleic acid molecule may extend up to 2.1 nm if fully stretched. For non-interacting dispersions of ferrite nanoparticles, and without consideration of any solvent–ligand interaction, a dense oleic acid ligand layer thickness of 1.4–1.6 nm is typically observed using SANS (Disch *et al.*, 2012 ▸). An interparticle separation distance as short as 2.5 nm therefore requires a certain degree of interpenetration of the oleic acid ligand shells.

Dense crystalline arrangements of the same nanoparticles obtained by a drop casting approach and reported in earlier work provide complementary information on the interparticle distances in long-range-ordered assemblies. For the nanospheres, a face-centred cubic packing was observed with a cubic lattice parameter of 17.5 (1) nm, corresponding to an interparticle distance of 12.4 (1) nm and an interparticle separation distance of 3.4 (1) nm (Disch *et al.*, 2013 ▸). The nanocubes preferentially organize into mesocrystals of body-centred tetragonal symmetry, with lattice parameters of *a* = 13.1 (1) nm and *c* = 17.8 (1) nm, corresponding to a nearest-neighbour distance of 13.1 (1) nm and a face-to-face distance of 4.6 (1) nm for the face-to-face direction (Disch *et al.*, 2011 ▸). The determined interparticle distances in concentrated ferrofluids of both spheres and cubes are therefore even shorter than those observed in the crystalline arrangements of the same particles. Considering the dimensions of the inorganic nanocubes (edge length 8.6 nm, space diagonal 10.2 nm), a face-to-face orientational alignment is very likely.

The correlation peak of the structure factor is directionally isotropic for both samples even in an applied field, *i.e.* appearing as a symmetric ring on the detector image, which indicates that there is no preferred orientation of the nearest-neighbour distances. In contrast to the nanospheres, however, the structure factor of the concentrated nanocube ferrofluid shown in Fig. 2 ▸ displays a clear field dependence of the correlation peak intensity and *Q* position, which will be analysed in greater detail below.

### 3.2. Field dependence of the structure factor

**Figure 3:**
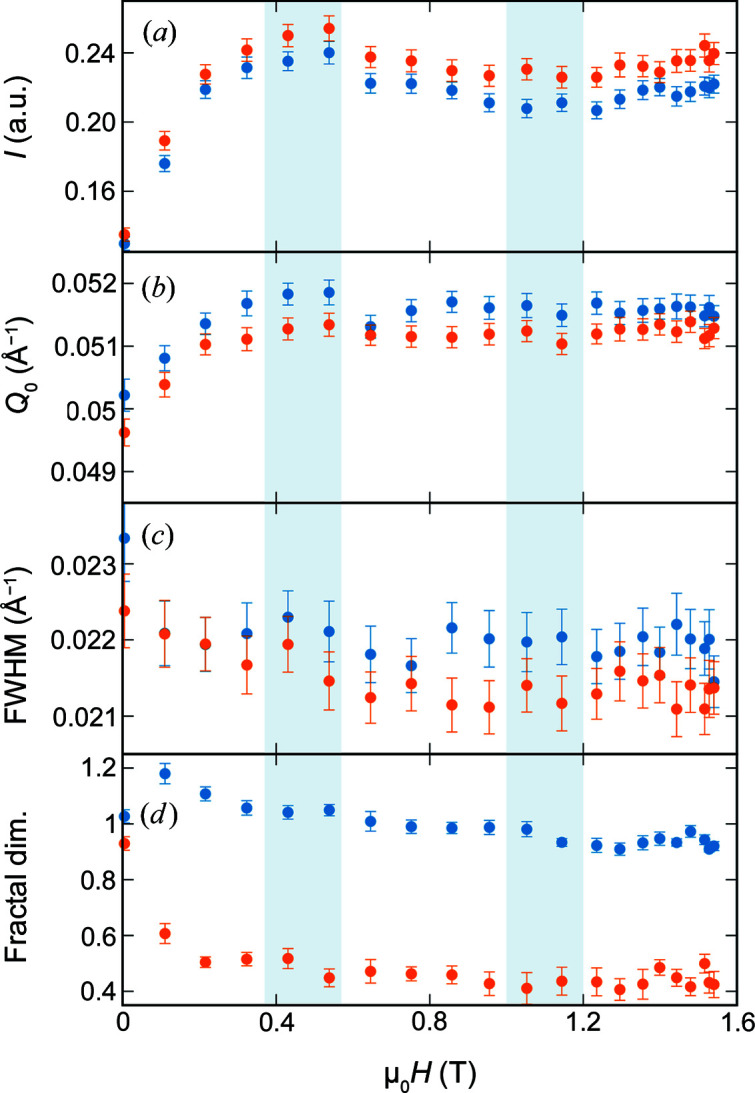
The field dependence of the nanocube structure factor including (*a*) the integrated intensity, (*b*) the *Q* position and (*c*) the FWHM of the correlation peak in the extracted structure factor, and (*d*) the fractal dimension of the scattering intensity in the low *Q* range. All parameters are displayed for the directions parallel (orange) and perpendicular (blue) to the applied field. Field ranges with maximum and minimum correlation peak intensity are indicated with blue–grey boxes as a guide to the eye.

The field dependence of the structure factor characteristics for the nanocubes is presented in Fig. 3 ▸. The integrated intensity of the correlation peak [Fig. 3 ▸(*a*)] increases significantly with applied field up to about 0.4 T. Interestingly, a slight decrease in the correlation peak intensity is observed, with a minimum near 1.1 T and a subsequent increase, reaching nearly the maximum intensity at the highest applied field of 1.54 T. The lower concentration of agglomerates associated with the intensity minimum might be attributed to a structural rearrangement and reorganization of the nanocubes. In addition, the correlation peak intensity is slightly stronger in the direction parallel to the applied field, indicating that there are more agglomerates in this *Q* direction. The position of the correlation peak [Fig. 3 ▸(*b*)] shifts to a larger *Q* with increasing field, reaching its maximum, and the maximum difference between the directions parallel and perpendicular to the applied field, near 0.4 T. In the larger field range, the *Q* position of the correlation peak perpendicular to the field direction decreases slightly, approaching the *Q* position parallel to the field direction in the highest applied field of 1.5 T. This corresponds to a slightly stronger compression of the interparticle distance in the perpendicular direction, from 12.5 nm in zero field to 12.1 nm near 0.4 T, compared with 12.2 nm in the field direction. At the same time, the FWHM of the correlation peak [Fig. 3 ▸(*c*)] decreases slightly with applied field. These observations support the picture of a field-induced structural rearrangement.

In addition to the variation in the interparticle correlation, we observe a directionally anisotropic scattering contribution in the lower *Q* range that indicates oriented attractive interparticle interactions. The field dependence of the fractal dimension is presented in Fig. 3 ▸(*d*). With increasing applied magnetic field, the fractal dimension measured perpendicular to the applied field direction decreases systematically from about 1.2 in a low field towards 1, indicating a stiffening from a coil-like structure into a straight linear shape of the nanocube agglomeration oriented into the field direction. At the same time, the fractal dimension measured parallel to the applied magnetic field nearly vanishes to about 0.4, which is very similar to the slope of the nanocube form factor in the low *Q* range (Fig. 2 ▸). We interpret this decrease in the fractal dimension as the stiffening of the mesoscopic nanoparticle arrangements from a flexible arrangement in zero field towards a linear agglomerate oriented parallel to the applied magnetic field.

We understand the observation of elongated aggregates oriented parallel to the applied field, while the correlation peak remains nearly isotropic, as follows. The concentrated nanoparticle dispersion must contain small domains of densely packed nanocubes (short interparticle distance) that are randomly oriented (isotropic correlation peak) and assembled into larger agglomerates which have an overall elongated shape (fractal dimension). This scenario is similar to the chain formation via cluster–cluster interactions recently reported for magnetic nanoparticles of comparable size (Myrovali *et al.*, 2021 ▸).

### 3.3. Analysis in real space

For a better understanding of the arrangement of nanospheres and nanocubes in real space, we derive the 2D pair correlation function from the SANS data using the truncated singular value decomposition (SVD) technique (Bender *et al.*, 2019 ▸). We focus on the field conditions with maximum and minimum contributions of the structure factor identified for the nanocubes in Fig. 3 ▸.

**Figure 4:**
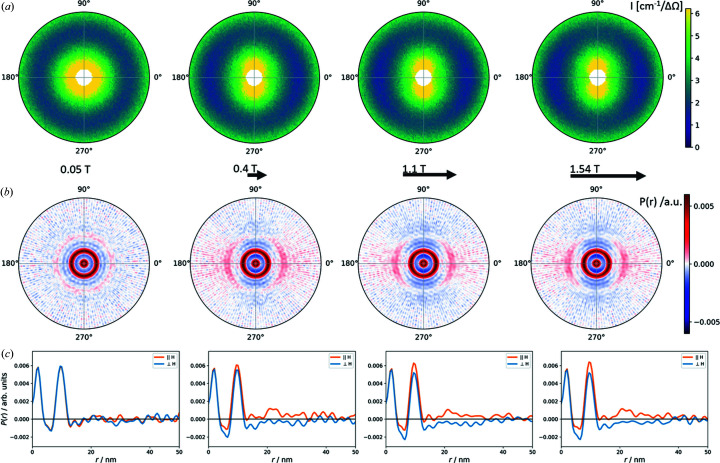
(*a*) SANS data with *Q* < 0.06 Å^−1^, (*b*) the 2D correlation function with *r* < 50 Å, and (*c*) the 1D correlation functions parallel (orange) and perpendicular (blue) to the applied field direction for the concentrated dispersion of spherical nanoparticles. The applied magnetic fields of 0.05, 0.4, 1.1 and 1.54 T (left to right) are indicated as horizontal arrows.

The field-dependent pair correlation function of the nanospheres is shown in Fig. 4 ▸. We observe a relatively weak short-range-order correlation with two maxima in the correlation function, corresponding to the self-correlation of the primary particle and the interaction distance to its nearest neighbours. Upon application of a magnetic field, this correlation remains nearly constant but reveals a slight variation in the angular dependence. The positive correlations (red) in the 2D pair correlation function [Fig. 4 ▸(*b*)] suggest a preferential particle organization primarily into the field direction. A direct comparison of the 1D pair correlation functions parallel and perpendicular to the field direction [Fig. 4 ▸(*c*)] illustrates the anisotropy in the nearest-neighbour correlation that is more probable in the field direction and reveals a third, weaker, correlation maximum in the field direction corresponding to second nearest-neighbour interactions at 21–22 nm distance.

**Figure 5:**
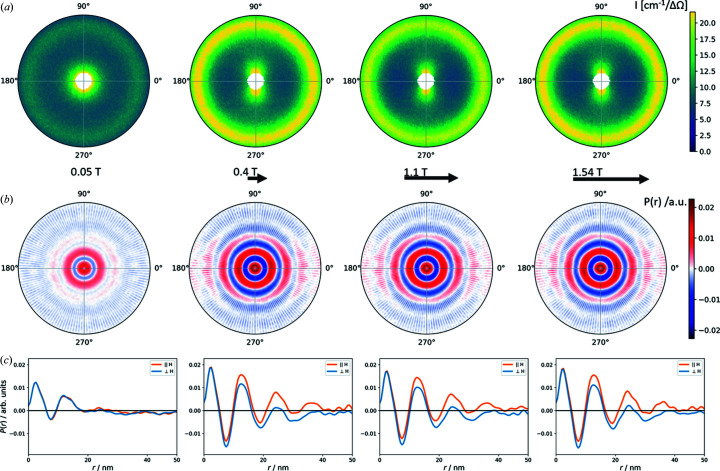
(*a*) SANS data with *Q* < 0.06 Å^−1^, (*b*) the 2D correlation function with *r* < 50 Å, and (*c*) the 1D correlation functions parallel (orange) and perpendicular (blue) to the applied field direction for the concentrated dispersion of nanocubes. The applied magnetic fields of 0.05, 0.4, 1.1 and 1.54 T (left to right) are indicated as horizontal arrows.

The 2D pair correlation function of the nanocubes (Fig. 5 ▸) is significantly stronger than that observed for the nanospheres and reveals much richer structural information. As in the case of the spherical particles, we observe two maxima in the pair correlation function in the near-zero-field condition. With the application of a magnetic field, the positive correlations in the 2D pair correlation function [Fig. 5 ▸(*b*)] are strongly enhanced in the field direction, corresponding to an elongation of the nanoparticle arrangement primarily into the field direction. In addition to the first nearest-neighbour correlation distance also visible perpendicular to the field direction, at least two more interaction distances (and potentially even more, suppressed by the limited *Q* resolution here) are present in the field direction. This underlines the strong directional anisotropy of the nanocube arrangement that was already observed from the structure factor in reciprocal space. Moreover, we note that the correlation maxima are separated by clearly negative correlations (blue), suggesting a very regular confined local packing of the nanocubes. The 1D pair correlation function [Fig. 5 ▸(*c*)] reveals that several pair correlation maxima may even be interpreted as overlap of two slightly different pair correlation distances. In the near-zero-field condition, the first nearest-neighbour correlation resembles a combination of two pair correlation distances of 11.8 nm and 13.7 nm. Overall, these are mixed into an average correlation distance of 12.6 nm that is in good agreement with the observation of the structure factor. Analogously, the third maximum of the correlation function that appears in an applied field, attributed to the second particle interaction shell, appears split into distances of 24 nm and 26.5 nm, *i.e.* nearly twice the first interaction distances. This observation appears reasonable in view of the more complex packing schemes that are possible for cubes as a direct consequence of their shape. For a body-centred packing of nanocubes, as is typically observed, the direct face-to-face distance may be very similar to, but distinguishable from, the corner–corner distance. The body-centred tetragonal mesocrystal structure reported for this sample of nanocubes (Disch *et al.*, 2011 ▸) corresponds to a face-to-face distance of 13.1 nm and a corner-to-corner distance of 12.8 nm, which are indeed very similar.

**Figure 6:**
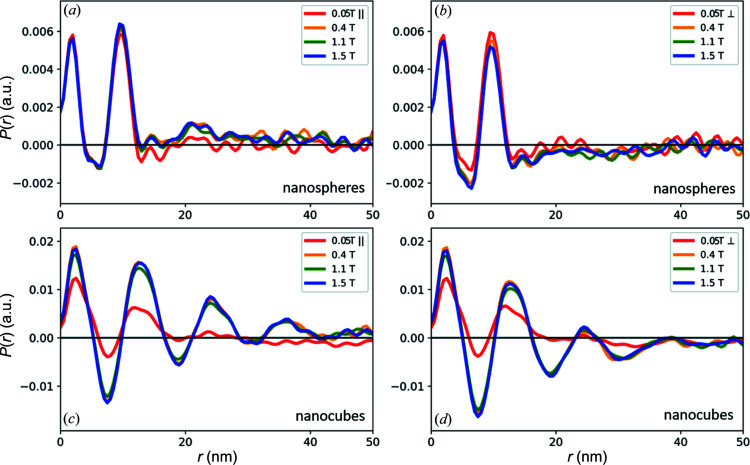
The field dependence of the 1D correlation function, (*a*), (*c*) parallel and (*b*), (*d*) perpendicular to the applied field for (*a*), (*b*) the nanospheres and (*c*), (*d*) the nanocubes.

The presentation of the 1D pair distance correlation functions in Fig. 6 ▸ illustrates the field dependence of the structure formation in both samples. For the nanospheres only a slight field dependence of the arrangement is observed in the appearance of a weak third correlation maximum in the field direction that is attributed to a second nearest-neighbour correlation. The second correlation maximum exhibits a systematic enhancement in the field direction and decreases perpendicular to it with applied field strength. The strong influence of the magnetic field on the structure formation of the nanocubes manifests in the significant enhancement of the overall pair correlation function with applied field. We observe a clear organization on the local scale, with positive and negative correlation probabilities indicating a regular order up to about 20 nm. Beyond 20 nm, the correlation function remains overall positive in the field direction and negative perpendicular to it, indicating the strong directional anisotropy of the overall distribution of nanoparticles. We further observe that the correlation intensity increases up to 0.4 T, then decreases up to 1.1 T, and then increases again up to the final applied field of 1.54 T. This corresponds directly to the field-dependent variation already observed for the structure factor. In addition, analysis of the 1D pair distance correlation functions provides detailed structural information through the resolution of overlapping correlation distances, and a slightly varying response to the applied field is observed.

We attribute the peculiar field-dependent behaviour of the nanocubes to a field-induced structure formation and re­organization in a concentrated dispersion and condense our observations into the following scenario.

The strong particle concentration leads to elongated aggregates of nanocubes already present in a very low magnetic field, which both grow and orient themselves with the applied magnetic field as is consistently observed in the fractal dimension and the 2D pair correlation function. On the basis of the very short interparticle interaction distances, we argue that the nanocubes must be orientationally aligned. This alignment fixes their orientation relative to each other and hinders free reorientation of the individual nanocubes, whereas the aggregate remains oriented in the direction of the applied field. We speculate that once the applied field is sufficiently strong to align the nanoparticle spins out of their easy magnetization direction, the local dipolar interactions within the nanoparticle arrangement are affected by a change in direction of the nanoparticle spins that is sufficient to introduce a slight structural rearrangement. This appears reasonable considering the macroscopic magnetization measurements of the non-interacting nanocubes, which exhibit the classical Langevin behaviour of superparamagnetic nanoparticles (Disch *et al.*, 2012 ▸). Around 0.4 T the ensemble is nearly saturated, indicating that precession of the nanoparticle spins around the field direction is still possible. However, near the maximum field applied here (1.54 T), spontaneous magnetization is clearly reached, *i.e.* atomic moments will be aligned along the field regardless of the orientation of the particle. A similar field-induced reorientation of the magnetic moments in a nanoparticle arrangement has been observed for magnetotactic bacteria (Orue *et al.*, 2018 ▸).

Considering the importance of ferrofluid stability for applications, it will be extremely interesting to verify the effect of such a seemingly subtle variation in nanoparticle shape on the magnetoviscous behaviour of concentrated ferrofluids, *e.g.* by using a combined magneto–rheo–SANS approach (Zákutná *et al.*, 2021 ▸).

## 4. Conclusions

We have revealed the impact of nanoparticle shape on structure formation in concentrated dispersions, *i.e.* ferrofluids, of maghemite nanoparticles. Dispersions of spherical and cuboidal nanoparticles of very similar particle size, magnetic moment and concentration exhibit a directionally isotropic short-range interaction potential on the local scale.

The nanocube ferrofluid reveals a much stronger interparticle correlation along with a strong field-induced directional anisotropy for larger length scales. We interpret these as large elongated arrangements that align parallel to an applied magnetic field and consist of short-range-ordered nanoparticles or smaller domains of locally ordered nanoparticles with isotropic orientation. The observed particle-to-particle distances are remarkably small, even compared with the crystalline arrangements of the same particles, and a preference for face-to-face orientation of the nanocubes is likely.

The distinct structure formation behaviour of the cuboidal nanoparticles is furthermore expressed in a much stronger correlation peak, which exhibits an unusual field dependence that we interpret as field-induced order and structural re­arrangement. Analysis of the two-dimensional pair correlation distribution additionally provides a clearer insight into the interparticle correlation distances, uncovering distinguishable nearest-neighbour distances resulting from close packing of the cuboidal nanoparticles.

The stability of ferrofluids is crucial for technological application, *e.g.* in magnetic seals or lubricants. Stronger interactions of faceted nanoparticles, as observed here, may become beneficial for an economic application if less material is required for similar structure formation effects.
